# Dynamics of Isomeric and Enantiomeric Fractions of Pinene in Essential Oil of *Picea abies* Annual Needles during Growing Season

**DOI:** 10.3390/molecules26082138

**Published:** 2021-04-08

**Authors:** Liucija Kamaitytė-Bukelskienė, Kristina Ložienė, Juozas Labokas

**Affiliations:** Nature Research Centre, Institute of Botany, Akademijos g. 2, LT-08412 Vilnius, Lithuania; liucija.bukelskiene@gamtc.lt (L.K.-B.); juozas.labokas@gamtc.lt (J.L.)

**Keywords:** Norway spruce, essential oil, α-pinene, β-pinene, (−) and (+) enantiomers

## Abstract

Norway spruce (*Picea abies* (L.) H. Karst.) is one of the most important commercial tree species distributed naturally in the Boreal and subalpine forest zone of Europe. All parts of spruce trees, including needles, accumulate essential oils with a variety of chemical properties and ecological functions, such as modulating plant–insect communication. Annual needle samples from 15 trees (five from each of three habitats) of 15–17 years old were assayed for essential oils and their major compounds, including α-pinene, β-pinene, (1S)-(−)-α-pinene, and (1R)-(+)-α-pinene across a growing season. Results showed strong positive correlation between percentages of α- and β-pinene isomers (r = 0.69, *p* < 0.05) and between pinene isomers and essential oils: α-pinene correlated with essential oil stronger (r = 0.62, *p* < 0.05) than β-pinene (r = 0.33, *p* < 0.05). Correlation analyses performed with some weather conditions, including average monthly temperature, growing sum of effective temperatures over 5 °C, duration of sunshine, accumulated precipitation, relative humidity, and pressure, showed that temperature is the most important weather condition related to pinene dynamics: negative correlations of moderate strength were established between percentages of α- and β- pinenes and average monthly temperatures (r = −0.36, *p* < 0.01, n = 75 and r = −0.33, *p* < 0.01, n = 75, respectively). Out of pinene enantiomers, only (1S)-(−)-α-pinene showed some negative correlation with monthly temperature (r = −0.26, *p* < 0.05, n = 75). Different patterns of essential oil and pinene dynamics during growing season within separate habitats suggested that some genetic variables of *Picea abies* might be involved.

## 1. Introduction

Norway spruce, *Picea abies* (L.) H. Karst. (Pinaceae), is a large essential oil-bearing evergreen coniferous tree species valued largely for timber production. It is the main species in the Boreal and subalpine conifer forests of Europe, from Central (in mountains) to Northern and Eastern Europe, up to the Ural Mountains [1].

*Picea abies* accumulates essential oils in all parts of the plant—in leaves (needles), wood, cones, and bark [2]. However, certain peculiarities exist regarding the quantitative and qualitative composition and distribution of essential oils across various parts of the plant. For example, it was reported that in *Picea abies* stem wood, higher percentages of α-pinene accumulate in heartwood than in sapwood, while β-pinene takes over in sapwood, compared to heartwood [3,4]. When comparing the yield of oil extractives from wood and bark of *Picea abies*, it was established that bark oil content exceeds that of wood by nearly three times [5]. A study on cone essential oil composition reported that α-pinene comprised 11%, and β-pinene amounted to 30% of the relative constitution of monoterpene fraction [6]. Monoterpenes constitute the biggest part of *Picea abies* essential oils, amounting to about 70%, with limonene, camphene, and pinene dominating often [7,8,9]. Most of the monoterpenes are good information carriers over long distances because they present low molecular weight lipophilic molecules; in general, a lot of ecologically important interactions between trees and their environment take place via the terpenes [10,11]. Thus, terpenes can perform signaling functions within the population by promoting the protective mechanisms of associated plants or adjacent plant tissues [11]. Monoterpene pinene is common in conifers and has two structural isomers, i.e., α-pinene and β-pinene with characteristic pine and turpentine smells, respectively [12,13]. Their biological functions and properties, although yet under-investigated, comprise quite a wide spectrum. A high therapeutic potential of pinenes with an emphasis on pharmacological activities of α- and β-pinene was revealed in a systematic review by Salehi et al. [14]. For example, α-pinene has been used as an antibiotic resistance modulator for a multidrug-resistant bacterium *Campylobacter jejuni* [15]. Additionally, α-pinene is an important terpenoid with anticancer properties, including against N2a neuroblastoma cells [16]. Synergistic anticancer effects have been demonstrated by α- and β-pinene against non-small cell lung carcinoma with paclitaxel [17]. The assessment of antimicrobial activity of α- and β-pinene against bacterial (*Escherichia coli*, *Staphylococcus aureus*, and *Bacillus cereus*) and yeast (*Candida albicans*) strains showed that (+)-β-pinene had 2–12 times higher activity than (+)-α-pinene against both Gram-positive and Gram-negative bacteria, as well as *C. albicans* [18]. Percentages of pinene isomers in essential oils vary considerably depending on species of *Picea* (Table 1); such a wide interspecific variation is subject largely to genetic differences between species, while the intraspecific variation may be predefined by genetic and environmental factors [19]. Most terpenes exist in pairs as enantiomers, and pinenes are not an exception—two pairs of enantiomeric forms of pinene isomers coexist naturally in nature: (−)-α-pinene and (+)-α-pinene, (−)-β-pinene and (+)-β-pinene [13,20]. It has been reported that needles of *Picea abies* accumulate higher amounts of (−)-α-pinene than xylem, while amounts of (−)-β-pinene are similar in both needles and xylem [21]. Persson et al. established that (+)-α-pinene is the second most prevalent monoterpene after (−)-β-pinene in stem and xylem of five-year-old twigs in *Picea abies* [22]. It has been suggested that differences in the enantiomeric composition of pinene isomers in plant tissues may be the result of alternative biosynthetic pathways operating to different extents in different tissues including needles [21].

The role of pinenes and some other terpenes are of utmost importance regarding conifer resistance against bark beetles and their associated fungi. In experiments with *Picea abies*, carried out in southern Sweden [23], it was established that terpene composition in stem bark may be related to the Norway spruce resistance against the European spruce bark beetle (*Ips typographus*) and bluestain fungus (*Ceratocystis polonica*). Moreover, the enantiomeric ratio of (−)-α-pinene was found significantly higher in trees that were attractive to the beetle, compared to unattractive trees [23], suggesting that this enantiomer is important for the life cycle of the beetle. In fact, it was reported earlier that the absence of (−)-α-pinene can slow down or completely prevent the biosynthesis of the bark beetle aggregation pheromone [21,24].

A study on receptor neuron discrimination of α-pinene and limonene enantiomers in the pine weevil (*Hylobius abietis*) reported that one olfactory receptor neuron showed a stronger response to the (+)-α-pinene than to (−)-α-pinene, while another neuron responded to limonene enantiomers vice versa. The study concluded that the enantiomeric ratios of volatile compounds may be important in host location by the pine weevil [25]. Studies of effects of methyl jasmonate-induced monoterpenes in Scots pine and Norway spruce tissues showed that (−)-β-pinene had a deterrent effect against *Hylobius abietis*, however, the effect depended on the (−)-β-pinene/(−)-α-pinene ratio being higher in pine than in spruce [26]. The common pine shoot beetle (*Tomicus piniperda*), which is reported to be the second most destructive shoot-feeding species in Europe [27], was most attracted to *Pinus nigra laricio*, emitting most (−)-α-pinene [28].

Some studies demonstrated that major changes in contents of terpenes occur in young leaves, with the developing needles being most strongly affected by age, and therefore, they differ markedly from mature needles in quantitative terpene composition [8]. In some plants, maximum yearly amounts were recorded during the first month from the start of bud growth with other maximums established in June and July [10]. Similar patterns of seasonal variations in amounts of most terpenes have been reported on white spruce (*p. glauca*) and blue spruce (*p. pungens*) [29,30]. A significant reduction in β-pinene, limonene, and α-phellandrene contents along with needle age from one to four years old has been reported for *Picea abies* [7]. In general, the terpenes in needles of *Picea abies* are controlled by genetic, physiological, and external factors. The most important factors seem to be genetic control, causing great differences between wild trees even at the same narrow location, and needle age, particularly during development [8].

The aim of this study was to establish seasonal dynamics of the quantitative composition of essential oils with the emphasis on α- and β-pinene and enantiomers of α-pinene in annual needles of *Picea abies*. To achieve this aim, we analyzed monthly changes in percentages of essential oils and pinene compounds in the same age (15–17 years old) trees in three very similar habitats, considered as replications. Correlation analyses between essential oil percentages and various weather conditions were employed to reveal this kind of relationship in seasonal dynamics of needle essential oils in *Picea abies*. The variation in the essential oil composition, particularly the contents of pinene isomers, is important to predict their biological effects, for example, to determine the most favorable or unfavorable periods for the associated insect pests.

**Table 1:** Percentages of α-pinene and β-pinene in essential oils of needles of different *Picea* species.

Species	α-Pinene, %	β-Pinene, %	Reference
*Picea abies*	27.7	5.4	[31]
*Picea abies*	9	2	[32]
*Picea abies*	14.16	4.75	[2]
*Picea engelmanni*	5.77	0.87	[2]
*Picea glauca*	15.85	–	[33]
*Picea glauca*	5.2	1.0	[29]
*Picea mariana*	16.62	–	[33]
*Picea omorika*	3.8 ^a^, 11.0 ^b^, 5.1 ^c^	0.7 ^a^, 0.9 ^b^, 0.6 ^c^	[34]
*Picea pungens*	4.06	–	[29]
*Picea pungens*	7.3	1.1	[30]
*Picea rubens*	10.36	3.63	[29]

^a^ habitat in Belgrade, ^b^ habitat in Hamburg, and ^c^ habitat in Zlatibor.

## 2. Results

### 2.1. Dynamics of Air-Dry Mass Yield and Quantitative Composition of Essential Oil in Annual Needles during the Growing Season

**Figure 1:**
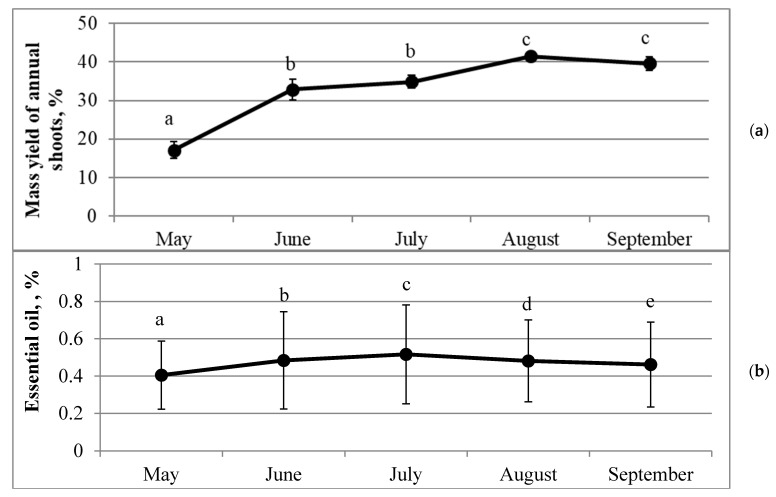
Dynamics of air-dry mass yield (**a**) and essential oil percentage (**b**) in *Picea abies* (n = 15) needles during the growing season. Error bars indicate ± SD; different letters denote statistically significant (*p* < 0.05) differences.

The dynamics of air-dry mass yield of annual shoots (n = 15 trees) during the growing season is demonstrated in Figure 1a and Table S1. The highest mass yield in August was 2.5 times bigger than the lowest one in May. The highest leap of growth was observed in the period from May to June (the mass has nearly doubled), while the lowest (only 2%) occurred in the period from June to July. A very small decrease was observed at the end of the growing season, from August to September. Most of the mean values of the mass yield differed significantly (*p* < 0.05) between months, except for those between June and July and between August and September.

**Figure 2:**
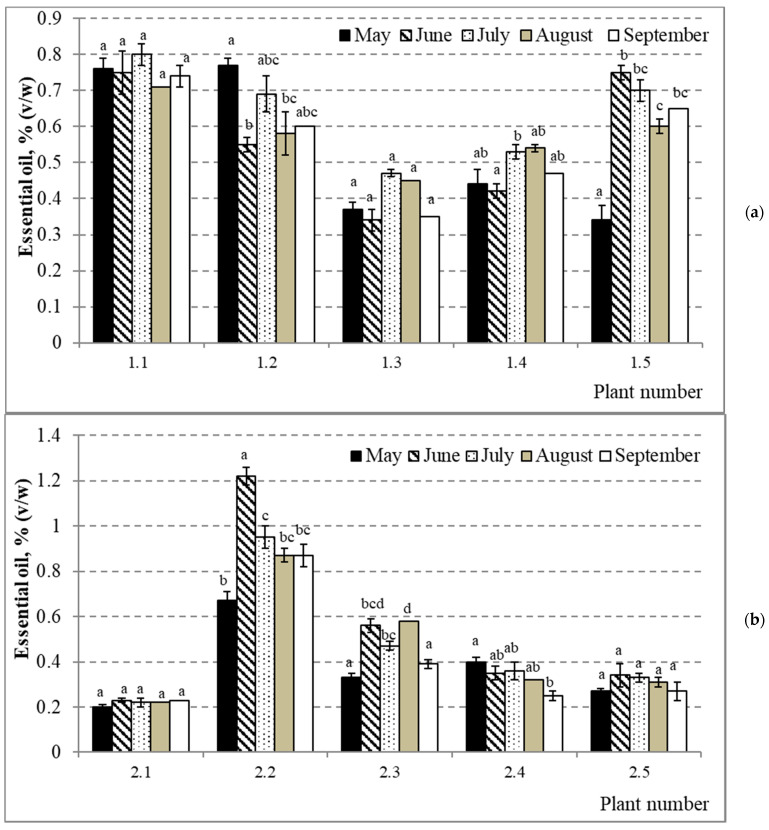

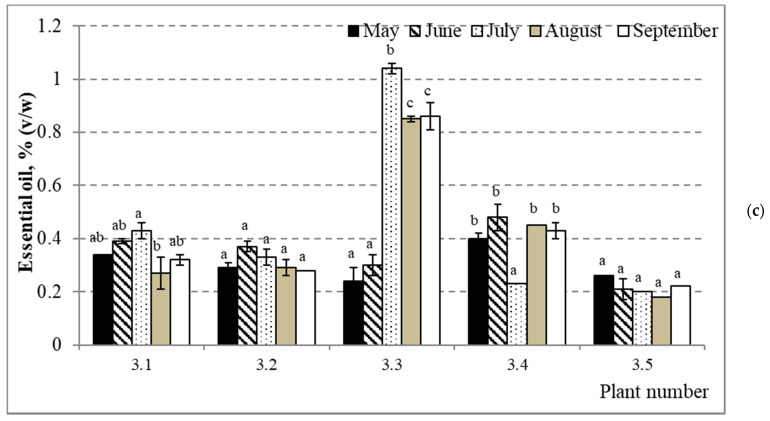
Variation of mean essential oil percentage (*v/w*) during the growing season in annual needles of individual plants of *Picea abies* in habitats No. 1 (**a**), No. 2 (**b**), and No. 3 (**c**). On X-axis, the first part of a combined number indicates habitat number, and the second indicates individual plant number within that habitat. Error bars indicate ± SD; different letters denote statistically significant (*p* < 0.05) differences between months within plants.

Percentages of essential oils varied with a range of 0.41–0.52% (*v/w*) on average during the growing season (Figure 1b, Table S1). However, no statistically significant differences were observed between months. The lowest average (0.41 ± 0.18% (*v/w*)) of essential oil percentage was established at the beginning of the growing season and the highest in July (0.52 ± 0.27% (*v/w*)). A decrease in essential oil percentage was observed in the second half of the growing season. However, *Picea abies* demonstrated different variation patterns of the quantitative composition of essential oils during the growing season, measured by the coefficient of variation (from CV = 4% in tree 1.1 to CV = 55% in tree 3.3), and percentages of essential oils differed significantly (*p* < 0.05) between months in most individuals of *Picea abies* (Figure 2a–c). For example, the highest percentages of essential oils were established in May, in June, in July, and in August in three, six, four, and two trees, respectively. Individuals No. 2.2 and No. 3.3 distinguished most, showing the highest percentages of essential oils (1.22 ± 0.04% (*v/w*) in June and 1.04 ± 0.02% (*v/w*) in July, respectively), significantly (*p* < 0.05), differing from the percentages in other months (Figure 2). While the lowest percentages of essential oils were established mostly at the beginning of the growing season (40% of trees in May), July was the month with the lowest percentage of essential oils in *Picea abies* No. 3.4 (Figure 2c). The amount of essential oils accumulated in this tree in July was significantly lower (*p* < 0.05) than in any other four months. One-way ANOVA revealed no significant differences between means of essential oil percentages between habitats across months except between habitats 1 and 3 (*p* = 0.048) in May. However, no significant effect of habitat was detected (*p* = 0.119). A bit different picture was obtained when pooled monthly data were compared between habitats, i.e., the significant differences were established between habitats 1 and 2 (*p* = 0.045) and 1 and 3 (*p* = 0.003). An estimated habitat effect in this case amounted to 11.8% (F = 4.825, *p* = 0.011).

### 2.2. Dynamics of Percentages of Pinene Isomers in Annual Needles during the Growing Season

Variations of α- and β-pinene percentages in essential oils of annual needles of all studied *Picea abies* trees are presented in Table S2. The results of correlation analysis showed a strong positive correlation between percentages of pinene isomers (r = 0.69, *p* < 0.05), implying that the syntheses of α- and β- isomers of pinene are quite closely linked to each other in *Picea abies*. Additionally, statistically significant positive correlations were established between percentages of pinene isomers and essential oils in *Picea abies* needles with α-pinene correlating to essential oil stronger (r = 0.62, *p* < 0.05) than β-pinene (r = 0.33, *p* < 0.05).

**Figure 3:**
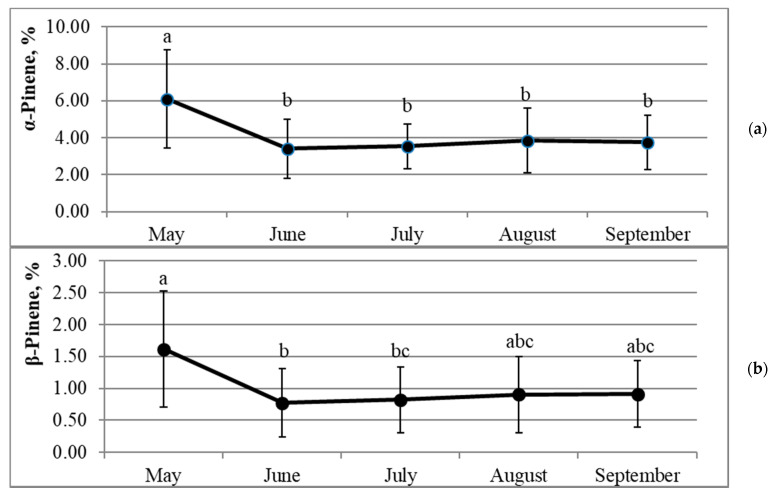
Dynamics of mean α-pinene (**a**) and β-pinene (**b**) percentages in essential oils of *Picea abies* (n = 15) needles during the growing season. Error bars indicate ± SD; different letters denote statistically significant (*p* < 0.05) differences.

The highest amounts of pinenes in annual needles of most investigated trees were found in May; only in three (2.1, 3.2, and 3.3) and two (1.2 and 3.3) individuals, the highest percentages of α-pinene and β-pinene, respectively, were found in other months (Table S2). The dynamics of mean percentages of both pinene isomers of the studied trees (n = 15) demonstrated two different trends (Figure 3a,b). The first trend—May through June—showed a very strong decrease in percentages of α- and β-pinenes: α-pinene decreased by 1.8 times (*p* = 0.009) and β-pinene by 2.1 times (*p* = 0.007). The second trend—June through August—demonstrated a statistically not significant increase in pinene isomers, i.e., by 12% for α-pinene and 18% for β-pinene (Figure 3a,b).

### 2.3. Dynamics of Percentages of Pinene Enantiomers in Annual Needles during the Growing Season

α-Pinene was determined to be enantiomerically pure (100%) for their (−) form or (−)-α-pinene exceeded (+)-α-pinene by 3 to 12.2 times (i.e., the racemic composition of α-pinene) (Table S3). The purity of 74.8% for the (1S)-(−) enantiomer in enantiomeric racemates of α-pinene was the lowest. Both enantiomers were interdependent, i.e., an increase in (1S)-(−) enantiomeric form in α-pinene fraction was always accompanied by a decrease in (1R)-(+) enantiomeric form (r = −0.95, *p* < 0.05). The percentage of (1R)-(+)-α-pinene weakly correlated with the percentages of essential oil and α-pinene (r = 0.29, *p* < 0.05 and r = 0.27, *p* < 0.05, respectively).

**Figure 4:**
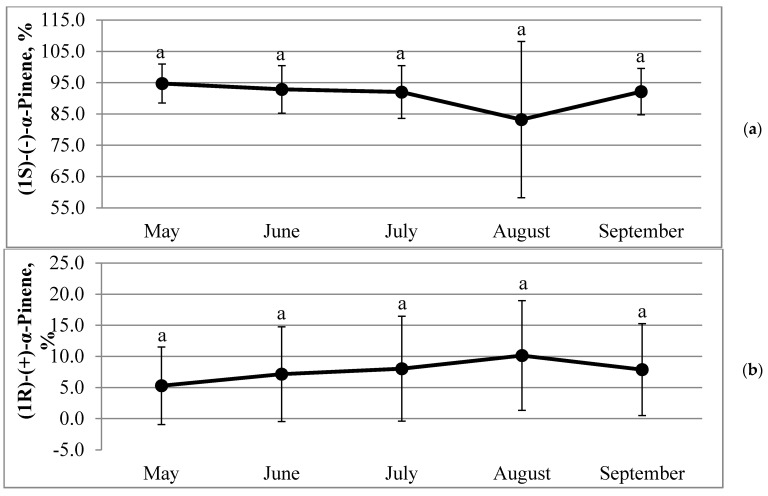
Dynamics of mean percentages of (1S)-(–) (**a**) and (1R)-(+) (**b**) enantiomers in an α-pinene fraction of essential oil of *Picea abies* (n = 15) needles during the growing season. Error bars indicate ± SD; different letters denote statistically significant (*p* < 0.05) differences.

The highest and the lowest percentages of (1S)-(−)-α-pinene in different *Picea abies* trees were established in different months, but two-thirds of the studied trees demonstrated their highest amounts of (1S)-(−)-α-pinene in May (Table S3). The dynamics of mean percentages of α-pinene enantiomers in α-pinene fraction (n = 15) demonstrated that (1S)-(−)-α-pinene was more or less stable from May to September, whereas (1R)-(+)-α-pinene increased from May to August (Figure 4a,b). However, regardless of the negative correlation between both enantiomers, the lowest percentage of (1S)-(−)-α-pinene occurred in July and the highest percentage of (1R)-(+)-α-pinene–in August. Both high standard deviation of (1R)-(+)-α-pinene established in May–September and that of (1S)-(−)-α-pinene observed in August resulted from the absence or undetectable quantities of these enantiomers in the respective months (Table S3).

### 2.4. Correlations with Weather Conditions

Pearson correlation analysis was employed to reveal correlations between the dynamics of the studied properties and changes of weather conditions of *Picea abies* growth environment. The results of correlation analysis with temperature are summarized in Table 2 and data on weather conditions are presented in Table 3.

Strong correlation was established between air-dry mass yield and sum of effective temperatures (≥5 °C) (r = 0.86, *p* < 0.01, n = 75). No correlations were established between the latter indicator and any those of essential oils, except for weak negative correlations with percentages of α-pinene (r = −0.286, *p* < 0.05, n = 75) and β-pinene (r = −0.252, *p* < 0.05, n = 75). Additionally, no correlation was established between air-dry mass yield and essential oil percentage during the growing season. Some weak correlations were established between the monthly accumulated precipitation and percentages of α-pinene (r = 0.313, *p* < 0.01, n = 75) and β-pinene (r = 0.295, *p* = 0.01, n = 75), of which the former was best pronounced within the habitat No. 1, where α-pinene correlated with precipitation quite strongly (r = 0.525, *p* < 0.01, n = 25). No correlations were established between the studied *Picea abies* properties and other weather conditions, such as humidity, pressure, and sunshine duration.

## 3. Discussion

The growing season of *Picea abies* in Lithuania starts in May and ends in September–October, depending on autumn weather conditions [35,36]. Our observations showed a continuous growth of annual shoots during the growing period. However, nonmonotonic mass change revealed the pattern of growth rate from May to August, with the fastest growth rate in May through June and a slightly decreasing growth rate from August to September (Figure 1; Table S1). Correlation analysis revealed a strong linear relationship between air-dry mass yield and average monthly temperature during the growing season (r = 0.77, *p* < 0.01, n = 75) (Table 2). Additionally, even stronger correlation was established between air-dry mass yield and sum of effective temperatures (≥5 °C) (r = 0.86, *p* < 0.01, n = 75). Only weak negative correlations established between sum of effective temperatures and percentages of α-pinene (r = −0.286, *p* < 0.05, n = 75) and β-pinene (r = −0.252, *p* < 0.05, n = 75), suggesting that this derivative meteorological indicator concedes to the use of temperature, a directly measurable factor. The absence of correlation between air-dry mass yield and essential oil percentage during the growing season may have some implications regarding the use of *Picea abies* needles as raw material for essential oil production. Regarding habitat effect on essential oil percentages, significant differences were established between habitats 1 and 2 (*p* = 0.045) and 1 and 3 (*p* = 0.003), suggesting that more fertile soil in habitat 1 could have discerned it out of the other two sites. However, an estimated habitat effect, in this case, amounted only to 11.8% (*p* = 0.011), which does not permit us to make any deeper inferences.

Essential oil percentages also demonstrated variation during the growing season—the lowest average of essential oil percentage was established in May (0.41%) and the highest in July (0.52%). These data correspond to those obtained in the geographical neighborhood of Lithuania—the percentage of essential oils in needles of *Picea abies* growing wild in Latvia varied from 0.36% to 0.53% and was lower in April–May [37,38]. However, the variation of essential oil percentages in individual trees during the growing season showed different patterns. The lowest percentages prevailed in the individual 3.5 (all months except May) and the highest percentages in the individual 2.2 (except for May and July) (Table S1). Thus, no significant correlation between essential oil percentage and the average monthly temperature was established (Table 2). Although different environmental conditions (climate, light conditions, soil chemistry) and harvesting time of raw material can influence the quantitative composition of essential oils [39], biosynthesis of volatile oils is a genetically controlled process. In our study, all individuals within a habitat grew under the same environmental conditions and thus were similarly influenced regarding their biosynthesis of essential oils. Therefore, some genetic variables of the studied trees might better explain differences in dynamics of essential oil percentages between them during the growing season. Some researchers argue that terpene composition varies less than the absolute amounts, thus allowing to discern *Picea abies* genotypes based on the variability of relative amounts of certain terpenes [10]. Based on relative amounts of limonene and myrcene, they distinguished two genotypic groups, namely, limonene > myrcene (group 1) and myrcene ≥ limonene (group 2). In our study, all 15 spruce trees had limonene dominating over myrcene, from 8 (tree 2.2) to 35.3 (tree 2.5) times on average (Table S4). Interestingly, both extreme limonene/myrcene ratios and two next to the extremes (trees 2.4 and 2.3) occurred in trees from the same habitat No. 2. Nevertheless, according to Schönwitz et al. (1989), only one genotype could be distinguished based on the limonene to myrcene ratio [19]. According to the researchers who studied odors of Norway spruce seedlings [40], spruce was defined as a bornyl acetate chemotype when its emission consisted of 35–54% bornyl acetate; 13–29% of limonene, and the content of every other compound was less than 10%. Only one tree (No. 2.1) met these conditions in our study, with 39.42% of bornyl acetate and 15.56% of limonene contents (Table S4).

The highest percentages of pinene stereoisomers α- and β- reached 9.32% and 3.11%, respectively (Table S2). Literature data suggest that percentage of α-pinene can vary from 14.2 to 27.7% in essential oils of needles of this conifer [2,31]. Other authors indicate that α-pinene can average to 10.67% of essential oil of *Picea abies* needles [41]. Meanwhile, β-pinene always has been found in amounts several times lower than those of α-pinene [31,41], although both isomers usually occur together in essential oils [2]. Our study demonstrated that an increase in the percentage of α-pinene was accompanied by an increase of β-pinene in essential oils of annual needles of *Picea abies* (r = 0.69, *p* < 0.05). Similar correlation patterns between these pinene isomers were also observed in cones and leaves of *Juniperus communis* with r = 0.44 (*p* < 0.05) and r = 0.50 (*p* < 0.05), respectively [42]. Therefore, it can be presumed that the syntheses of α- and β- isomers of this monoterpene are mutually dependent in *Picea abies* and in other conifers.

The highest amounts of pinenes in annual needles in May were followed by a sharp decrease in June (Figure 3). Negative correlations of moderate strength were established between the percentages of α- and β- pinenes and average monthly temperatures (r = −0.36, *p* < 0.01, n = 75 and r = −0.33, *p* < 0.01, n = 75, respectively) (Table 2). The highest percentages of α-pinene and β-pinene in needles of *Picea abies* growing in Latvia during this period (May–September) also were established in May (0.67% and 0.47%, respectively); however, the amounts of both isomers went down gradually, reached the lowest percentages (0.39% and 0.15%, respectively) only in July, and began to rise again just in August [38]. Similar consistent patterns have been found by the studies on *p. sitchensis* and *p. pungens*—percentages of pinenes rose in essential oils of needles in May and then declined during the growing season [30,43].

According to the Lithuanian dendrologists, the growth of new *Picea abies* shoots in Lithuania starts in May [36,44]. A widely accepted concept exists that the onset of the growing season of *Picea abies* estimated by bud burst depends on the growing degree days above a certain threshold temperature, usually 5 °C [45,46,47,48]. However, one of the latest studies showed that buds of three spruce species (including *Picea abies*) were sensitive to frost probability for early phenological stages, whereas growing degree days controlled the remaining stages [49]. *Picea abies*, especially young 15- to 20-year-old trees, suffer from larvae of needle-eating pest *Lygaeonematus abietinus* Christ. (Tenthredinidae) almost every year, usually in May [36,50]. The larvae of *L. monacha* are also active in May when the air temperature reaches 10–15 °C [51,52]. Thus, the sprouting time of *Picea abies* needles coincides with the larvae stage of needle-eating pests *L. abietinus* and *L. monacha*. Most of the terpenes found in essential oils of plants have been demonstrated to possess insecticidal and repellent activities [53]. Although less research data is available on the effects of pinene as a repellent, compared to many other terpenes, this bicyclic monoterpene has also been shown to repel *Aedes aegypti*, *Aedes albopictus*, and *Musca domestica* [54,55,56]. Therefore, noticeably higher percentages of pinene in essential oils of annual needles at the beginning of the growing season, compared to the further growth period, may relate to plant defense against *L. abietinus* larvae. A study on the effects of *Ceratocystis polonica* on *Picea abies* showed significantly higher contents of terpenes in less susceptible and less damaged trees, compared to those more damaged 30 days after the inoculation [57]. Therefore, the increased amount of diterpenes can be considered a defensive response to the fungal infection. This may also indicate a possible relationship between the monoterpene contents and resistance of *Picea abies* to some biotic stress. As reported in one of the reviewed sources, needles of healthier-looking trees contain higher amounts of monoterpenes [7].

Each of pinene isomers has two enantiomeric forms: α-pinene exists as (1R)-(+)-α-pinene and (1S)-(–)-α-pinene and β-pinene as (1R)-(+)-β-pinene and (1S)-(–)-β-pinene. The enantiomeric composition of α-pinene is variable in time. Regardless of the month, (–)-α-pinene dominated in α-pinene fractions of all investigated essential oils. There was a very strong negative correlation between α-pinene negative and positive enantiomeric forms (r = −0.95, *p* < 0.05). Moreover, it was reported that in needle samples, (–)-α-pinene dominated over its (+) enantiomer [22,58,59]. It was also established that the spruce emissions into Boreal forest ambient air were dominated by (−)-enantiomer confirming that this enantiomer is prevalent over (+)-enantiomer in *Picea abies* [60]. Some data exist suggesting that these enantiomers possess different biological activities. For example, our previous research showed that different enantiomeric compositions of α-pinene have different effects on microorganisms—α-pinene with the enantiomeric composition of S < R inhibited the growth of bacteria *Staphylococcus aureus, Escherichia coli*, and *Candida* yeasts more strongly, while α-pinene with enantiomeric composition S ≈ R did so on the growth of *Aspergillus fumigatus, A. flavus, Trichophyton rubrum*, and *T. mentagrophytes* [61].

In our study, the highest percentage of (1S)-(–)-α-pinene was established in May (Table S3). It was reported that the enantiomeric composition in *Picea abies* change from more (−)-α-pinene in the younger parts to more (+) enantiomer in the older parts [22]. In our study, higher percentages of (1S)-(–)-α-pinene in May, in comparison to other months, correspond to the reported observation. Regardless of the negative correlation between both enantiomers of α-pinene, the lowest percentage of (1S)-(–)-α-pinene and the highest percentage of (1R)-(+)-α-pinene were observed in different months. It could be related to the established fact that monoterpene-producing enzymes (i.e., monoterpene synthases) are enantiomer specific [62]. Effects of environmental factors could not be excluded as well. For example, it has been reported that biosynthesis of (−)-α-pinene is dependent on light levels, which either activate the synthase enzymes and/or provide the necessary energy for biosynthesis [60]. However, in our study, correlation analyses between monthly sunshine duration and amounts of any of pinene enantiomers, isomers, or total essential oils revealed no correlation. This result could be influenced by daily photoperiodism and the angle of sunrise. For example, the longest sunshine duration, 350 h, occurred in August (Table 3), when days were shorter, compared to June by more than 2 h on average, and sunrise was correspondingly lower than its zenith in June. Some weak correlations were established only between the monthly accumulated precipitation and percentages of α-pinene (r = 0.313, *p* < 0.01, n = 75) and β-pinene (r = 0.295, *p* = 0.01, n = 75), of which the former was best pronounced within the habitat No.1, where α-pinene correlated with precipitation quite strongly (r = 0.525, *p* < 0.01, n = 25).

Our results show that different essential oil chemotypes of *Picea abies* could be found under different growing and environmental conditions. Different chemical compositions can considerably affect the biological activity of the essential oil, including the antimicrobial activity, as revealed with thyme (*Thymus vulgaris* L.) extracts [63,64]. It is evident that a better knowledge of seasonal dynamics of pinene isomers and, particularly their chiral compounds, is increasingly important in revealing insect–plant or plant–plant communication in future warmer climate conditions since insects and plants discriminate and respond to changes in chiral compounds [60]. Our study showed that both intrinsic and environmental factors predefine the dynamics of isomeric and enantiomeric fractions of pinene in essential oils of *Picea abies* annual needles during the growing season. Different patterns of essential oil dynamics during the growing season within separate habitats suggested that some genetic variables of *Picea abies* might be involved. Therefore, further research is needed to justify factors causing chemotypic variation in *Picea abies* better. This could be best achieved by extending the studies on the chemical composition of natural extracts and their biological activities, including natural antioxidants and antimicrobial compounds [65].

## 4. Materials and Methods

### 4.1. Plant Material and Habitats

Three *Picea abies* habitats of anthropogenic origin were chosen in the Vilnius region (Table 4).

Five individual plants of 15–17 years old were chosen randomly and marked in each habitat; in total, 15 individual plants were studied. Each plant was assigned a number by combining habitat number with plant number within that habitat, i.e., 1.1, 1.2, 1.3, 1,4, 1.5, 2.1, 2.2, 2.3, 2.4, 2.5, 3.1, 3.2, 3.3, 3.4, 3.5. Annual shoots were sampled from each *Picea abies* tree separately with monthly intervals from May to September, on day 28 or 29 of each month. The period of sampling was chosen considering the duration of the growing season of *Picea abies* in Lithuania, where its growth begins in the middle-end of May and lasts 4–5 months [35]. Annual shoots were collected at a height of 1.5–2.5 m from all sides of each plant. Shoot samples were then dried at room temperature separately. To establish air-dry mass, shoots were weighed before and after drying. The yield of air-dry mass was then calculated by the following formula: Y = C/D*100%, where Y–air-dry mass yield (%); C–mass of air-dry shoots (g), D–mass of fresh shoots (g).

### 4.2. Isolation and Analysis of Essential Oils

Essential oils were isolated from air-dry needles (free from stems) by hydrodistillation in Clevenger-type apparatus (Simax) for two hours [66]. Distillation of essential oils of each sample was carried out in three replications, and the essential oil of the same sample was collected in the same bottle. The content of essential oil was calculated in % (*v/w*), based on the weight of air-dry mass of needles.

Afterward, 1% solutions of essential oils were prepared with a mixture of diethyl ether and n-pentane (1:1) for further investigations. The analysis of essential oils was carried out using a FOCUS GC (Thermo Scientific, Waltham, MA, USA) gas chromatograph with a flame ionization detector (FID). Data were processed with the CHROM-CARD S/W. The silica capillary column TR-5 (30 m, i. d. 0.25 mm, film thickness 0.25 µm) was used for the analysis of α and β pinenes, myrcene, limonene, camphene, and bornyl acetate with the following GC parameters: carrier gas helium flow rate 1.6 mL/min; temperature program from 40 °C to 250 °C increasing at 4 °C/min; detector temperature 260 °C; split injector was heated at 250 °C. The identification of α and β pinenes, myrcene, limonene, camphene, and bornyl acetate was carried out by the comparison of the retention time (RT) of their GC peaks in the FID chromatograms with the RT of α and β pinenes, myrcene, limonene, camphene, and bornyl acetate analytical standards (Sigma-Aldrich, St. Louis, MI, USA) under the same GC parameters and column. The percentage amounts of these compounds were recalculated according to the areas of the FID chromatographic peaks assuming that all constituents of the essential oil comprise 100%.

α-Pinene enantiomers were separated on HP^–^Chiral–20B column (30 m length, 0.249 mm id, 0.25 µm film thickness) (Figure S1). The following GC parameters were used for the analysis of enantiomers: carrier gas helium flow rate of 1.6 mL/min; temperature program from 85 to 160 °C increasing at 5 °C/min; detector temperature 260 °C; split injector was heated at 250 °C. The identification of (1R)-(+)-α-pinene and (1S)-(–)-α-pinene was carried out by the comparison of RT of its GC peaks in FID chromatograms with the RT of (1R)-(+)-α-pinene and (1S)-(–)-α-pinene analytical standards (Sigma-Aldrich; purity (GC area %) ≥98.5% and ≥99.0%, respectively) under the same GC parameters and column. The percentage amounts of enantiomers were recalculated according to the areas of the FID chromatographic peaks assuming that the monoterpene α-pinene fraction is 100%.

### 4.3. Meteorological Data

Weather data for the Vilnius region were obtained from the Lithuanian Hydrometeorological Service (sunshine duration, accumulated precipitation, and mean daily temperatures for the sum of effective temperatures) and from Timeanddate.com [67] (Table 3).

### 4.4. Statistical Analysis

Statistical data processing, including calculation of means, standard errors, standard deviations, coefficients of variation (CV), Spearman’s rank correlation coefficients (r), the Kruskal–Wallis test, and probabilities (*p*), was carried out with STATISTICA^®^ 7 and MS Excel. One-way ANOVA with Kruskal–Wallis test was used for the assessment of quantitative changes of essential oils in individual plants during the growing season. Pearson correlation analyses were performed with SPSS Statistics and ANOVA (comparison of means and GLM univariate analysis) used for statistical evaluation of habitat effects.

## Figures and Tables

**Table 2:** Pearson correlations between the studied properties of *Picea abies* annual needles and average monthly air temperature (°C) of their growth environment.

Property	Correlation Coefficient R	Significance *p*	Sample Size N
Air-dry mass yield, g:			
all habitats (by all trees)	0.773 **	<0.01	75
all habitats (by avg. tree)	0.786 **	<0.01	15
habitat 1 (by all trees)	0.733 **	<0.01	25
habitat 2 (by all trees)	0.817 **	<0.01	25
habitat 3 (by all trees)	0.782 **	<0.01	25
habitat 1 (by avg. tree)	0.751 ns	0.144	5
habitat 2 (by avg. tree)	0.827 ns	0.084	5
habitat 3 (by avg. tree)	0.792 ns	0.110	5
all habitats jointly	0.791 ns	0.111	5
Essential oil, %:			
all habitats (by all trees)	0.126 ns	0.282	75
all habitats (by avg. tree)	0.311 ns	0.259	15
habitat 1 (by all trees)	0.129 ns	0.540	25
habitat 2 (by all trees)	0.133 ns	0.526	25
habitat 3 (by all trees)	0.148 ns	0.479	25
habitat 1 (by avg. tree)	0.560 ns	0.326	5
habitat 2 (by avg. tree)	0.612 ns	0.273	5
habitat 3 (by avg. tree)	0.620 ns	0.264	5
all habitats jointly	0.762 ns	0.134	5
α-Pinene, %:			
all habitats (by all trees)	−0.355 **	0.002	75
all habitats (by avg. trees)	−0.544 *	0.036	15
habitat 1 (by all trees)	−0.573 **	0.003	25
habitat 2 (by all trees)	−0.367 ns	0.071	25
*habitat 3 (by all trees)	−0.233 ns	0.262	25
habitat 1 (by avg. tree)	−0.700 ns	0.188	5
habitat 2 (by avg. tree)	−0.726 ns	0.165	5
habitat 3 (by avg. tree)	−0.622 ns	0.263	5
all habitats jointly	−0.720 ns	0.169	5
β-Pinene, %:			
all habitats (by all trees)	−0.334 **	0.003	75
all habitats (by avg. trees)	−0.619 *	0.014	15
habitat 1 (by all trees)	−0.318 ns	0.122	25
habitat 2 (by all trees)	−0.321 ns	0.117	25
habitat 3 (by all trees)	−0.413 *	0.040	25
habitat 1 (by avg. tree)	−0.667 ns	0.219	5
habitat 2 (by avg. tree)	−0.762 ns	0.134	5
habitat 3 (by avg. tree)	−0.760 ns	0.136	5
all habitats jointly	−0.740 ns	0.156	5
(1S)-(–)-α-Pinene, %:			
all habitats (by all trees)	−0.261 *	0.024	75
all habitats (by avg. tree)	−0.547 *	0.035	15
habitat 1 (by all trees)	0.058 ns	0.784	25
habitat 2 (by all trees)	−0.297 ns	0.149	25
habitat 3 (by all trees)	−0.397 *	0.049	25
habitat 1 (by avg. tree)	0.327 ns	0.592	5
habitat 2 (by avg. tree)	−0.880 *	0.049	5
habitat 3 (by avg. tree)	−0.789 ns	0.113	5
all habitats jointly	−0.641 ns	0.244	5
(1R)-(+)-α-Pinene, %			
all habitats (by all trees)	0.190 ns	0.103	75
all habitats (by avg. tree)	0.391 ns	0.150	15
habitat 1 (by all trees)	−0.058 ns	0.784	25
habitat 2 (by all trees)	0.297 ns	0.149	25
habitat 3 (by all trees)	0.323 ns	0.116	25
habitat 1 (by avg. tree)	−0.327 ns	0.592	5
habitat 2 (by avg. tree)	0.880 *	0.049	5
habitat 3 (by avg. tree)	0.858 ns	0.063	5
all habitats jointly	0.928 *	0.023	5

*—Correlation is significant at the 0.05 level (2-tailed); **—Correlation is significant at the 0.01 level (2-tailed); ns—Correlation is not significant.

**Table 3:** Monthly averages of weather indicators for the Vilnius region during the study period of 2015.

Indicator	May	June	July	August	September
Temperature, °C	11	16	17	20	14
Sum of effective temperatures (≥5 °C) *	295	611	993	1454	1717
Sunshine duration, h	220.0	291.1	235.9	350.0	146.3
Humidity, %	73	65	71	60	81
Pressure, mbar	1014	1017	1012	1020	1018
Precipitation, mm	79.6	16.1	67.9	25.2	55.0

* Sum of effective temperatures calculated from 1 January, but effectively from 8 March, the first day with the average daily temperature higher than 5 °C.

**Table 4:** Habitat data of *Picea abies* sampling sites (provided by the State Forest Enterprise).

Habitat No.	Habitat Type *	Age of Trees (Years)	Locality	WGS–84 Coordinates
1	Ndp	15	Sužionys	54.977820 N 25.469426 E
2	Ncl	15	Žemaitėliai	54.954513 N 25.327365 E
3	Ncl	17	Adomciškės	54.810057 N 24.987477 E

* Forest habitat types: Ndp–normal moisture (N), very fertile soil (d) on binary bedrock (*p*); Ncl –normal moisture (N), fertile soil (c) on light bedrock (l).

## Data Availability

Supplemental data are provided in Tables S1–S4 and Figure S1.

## Supplementary Materials

The following are available online, Table S1: Air-dry mass yield of annual shoots and contents of essential oils in leaves of *Picea abies* observed in May through September. In Plant # column, the first numeral stands for habitat number (as described in Materials and Methods), and the second stands for individual plant number within that habitat (as in Figure 2); SD –standard deviation; CV –coefficient of variation, Table S2: Variation of percentages of pinene isomers in the essential oil of *Picea abies* leaves observed in May through September. In Plant # column, the first numeral stands for habitat number (as described in Materials and Methods), and the second stands for individual plant number within that habitat (as in Figure 2); SD –standard deviation; CV –coefficient of variation, Table S3: Variation of ratio percentages of (1S)-(–) and (1R)-(+) enantiomers in the α-pinene fraction of *Picea abies* leaves observed in May through September. In Plant # column, the first numeral stands for habitat number (as described in Materials and Methods), and the second stands for individual plant number within that habitat (as in Figure 2); SD –standard deviation; CV –coefficient of variation, Table S4: Variation of myrcene, limonene, camphene, and bornyl acetate percentages in the essential oil of *Picea abies* leaves during the growing season, May–September. In Plant # column, the first numeral stands for habitat number (as described in Materials and Methods), and the second stands for individual plant number within that habitat (as in Figure 2); SD –standard deviation, Figure S1: Chiral-phase capillary GC analysis of (1S)-(–) and (1R)-(+) enantiomers of α-pinene in *Picea abies* (essential oil of individual plant No.1.1 in August).
