# Supplementary material for: Dynamics of Isomeric and Enantiomeric Fractions of Pinene in Essential Oil of Picea abies Annual Needles during Growing Season
Source: Molecules. 2021 Apr 8;26(8):2138. doi: 10.3390/molecules26082138 (PMC8068203; doi:10.3390/molecules26082138)

Table S1. Air-dry mass yield of annual shoots and contents of essential oils in leaves of *Picea abies* observed in May through September. In Plant # column, the first numeral stands for habitat number (as described in Materials and Methods), the second – for individual plant number within that habitat (as in Figure 2); SD – standard deviation; CV – coefficient of variation.

	Air-Dry Mass Yield of Annual Shoots (%)								Contents of Essential Oils (%)							
Plant #	May	June	July	Aug.	Sep.	Mean	SD	CV	May	June	July	Aug.	Sep.	Mean	SD	CV
1.1	19.15	36.96	35.59	42.56	42.60	35.37	9.61	27	0.76	0.75	0.80	0.71	0.74	0.75	0.03	4
1.2	15.64	25.89	33.63	41.77	42.37	31.86	11.30	35	0.77	0.55	0.69	0.58	0.60	0.64	0.09	14
1.3	17.50	34.18	33.65	41.25	39.98	33.31	9.47	28	0.37	0.34	0.47	0.45	0.35	0.40	0.06	15
1.4	16.95	35.50	34.65	40.48	39.80	33.48	9.59	29	0.44	0.42	0.53	0.54	0.47	0.48	0.05	11
1.5	17.29	34.92	36.74	42.25	41.68	34.58	10.16	29	0.34	0.75	0.70	0.60	0.65	0.61	0.16	26
2.1	15.65	34.05	35.63	42.75	39.39	33.49	10.53	31	0.20	0.23	0.22	0.22	0.23	0.22	0.01	6
2.2	14.92	30.54	35.36	40.97	36.97	31.75	10.12	32	0.67	1.22	0.95	0.87	0.87	0.92	0.20	22
2.3	12.03	31.15	34.00	40.07	37.47	30.94	11.10	36	0.33	0.56	0.47	0.58	0.39	0.47	0.11	23
2.4	18.09	32.40	33.56	41.83	37.91	32.76	9.01	28	0.40	0.35	0.36	0.32	0.25	0.34	0.06	17
2.5	17.02	30.62	33.19	39.97	36.53	31.47	8.81	28	0.27	0.34	0.33	0.31	0.27	0.30	0.03	11
3.1	16.96	31.57	35.12	40.98	40.60	33.05	9.81	30	0.34	0.39	0.43	0.27	0.32	0.35	0.06	18
3.2	17.55	33.70	33.65	42.75	39.80	33.49	9.74	29	0.29	0.37	0.33	0.29	0.28	0.31	0.04	12
3.3	18.95	35.08	33.46	41.28	40.35	33.82	8.96	26	0.24	0.30	1.04	0.85	0.86	0.66	0.36	55
3.4	18.09	32.86	36.25	40.89	39.06	33.43	9.10	27	0.40	0.48	0.23	0.45	0.43	0.40	0.10	25
3.5	21.42	34.36	38.98	42.07	39.22	35.21	8.19	23	0.26	0.21	0.20	0.18	0.22	0.21	0.03	14

Table S2. Variation of percentages of pinene isomers in essential oil of *Picea abies* leaves observed in May through September. In Plant # column, the first numeral stands for habitat number (as described in Materials and Methods), the second – for individual plant number within that habitat (as in Figure 2); SD – standard deviation; CV – coefficient of variation.

Plant #	α-Pinene (%)								β-Pinene (%)							
	**May**	**June**	**July**	**Aug.**	**Sep.**	**Mean**	**SD**	**CV**	**May**	**June**	**July**	**Aug.**	**Sep.**	**Mean**	**SD**	**CV**
1.1	9.32	4.90	4.32	2.72	4.30	5.11	2.49	49	0.47	0.30	0.28	0.23	0.31	0.32	0.09	28
1.2	8.51	4.54	4.21	5.20	5.55	5.60	1.71	31	1.62	1.59	1.81	1.91	2.02	1.79	0.19	11
1.3	6.27	4.11	3.76	6.27	3.40	4.76	1.40	29	1.84	0.90	0.84	1.30	0.81	1.14	0.44	39
1.4	8.45	1.63	3.62	4.96	5.22	4.78	2.50	52	1.92	0.26	0.58	0.66	0.71	0.82	0.64	78
1.5	6.38	3.47	4.41	5.04	5.77	5.02	1.14	23	2.73	0.89	1.08	1.34	1.46	1.50	0.72	48
2.1	2.53	2.61	1.25	1.95	1.58	1.98	0.59	30	1.20	1.03	0.62	0.81	0.85	0.90	0.22	24
2.2	8.63	6.70	5.16	5.04	5.70	6.24	1.48	24	3.11	2.00	1.76	1.65	1.89	2.08	0.59	28
2.3	4.98	3.99	3.89	4.07	3.83	4.15	0.47	11	1.40	0.64	0.64	0.70	0.78	0.83	0.32	39
2.4	6.64	3.60	3.82	5.69	2.88	4.53	1.57	35	2.25	1.29	1.49	1.85	1.14	1.60	0.45	28
2.5	6.54	4.07	3.11	1.38	3.20	3.66	1.88	51	0.77	0.41	0.43	0.15	0.43	0.44	0.22	50
3.1	7.59	0.94	4.24	3.22	1.39	3.48	2.66	76	2.80	0.24	0.97	0.74	0.40	1.03	1.03	99
3.2	2.05	4.25	4.47	3.39	3.53	3.54	0.95	27	0.66	0.54	0.42	0.46	0.51	0.52	0.09	17
3.3	1.34	1.35	3.87	4.74	4.20	3.10	1.63	53	0.32	0.31	0.80	0.91	0.97	0.66	0.32	49
3.4	8.77	3.93	2.18	4.09	4.00	4.59	2.46	54	2.42	0.90	0.46	0.88	0.90	1.11	0.76	68
3.5	3.26	1.14	0.86	–	1.66	1.38	1.21	88	0.75	0.29	0.16	–	0.47	0.33	0.29	88

Table S3. Variation of ratio percentages of (1S)-(–) and (1R)-(+) enantiomers in α-pinene fraction of *Picea abies* leaves observed in May through September. In Plant # column, the first numeral stands for habitat number (as described in Materials and Methods), the second – for individual plant number within that habitat (as in Figure 2); SD – standard deviation; CV – coefficient of variation.

Plant #	(1S)-(–)-α-Pinene (%)								(1R)-(+)-α-Pinene (%)							
	May	June	July	Aug.	Sep.	Mean	SD	CV	May	June	July	Aug.	Sep.	Mean	SD	CV
1.1	83.5	87.9	86.5	88.1	87.9	86.8	0.9	1	16.5	12.1	13.5	11.9	12.1	13.2	0.9	7
1.2	92.4	91.4	90.7	91.0	90.6	91.2	0.3	0.3	7.6	8.6	9.3	9.0	9.4	8.8	0.3	3
1.3	89.8	80.5	80.1	79.9	80.4	82.1	1.9	2	10.2	19.5	19.9	20.1	19.6	17.9	1.9	11
1.4	86.6	100	87.3	87.2	86.3	89.5	2.6	3	13.4	0	12.7	12.8	13.7	10.5	2.6	25
1.5	92.3	90.6	100	100	90.2	94.6	2.2	2	7.7	9.4	0	0	9.8	5.4	2.2	41
2.1	100	100	100	74.8	100	95.0	5.0	5	0	0	0	25.2	0	5.0	5.0	100
2.2	86.4	85.8	86.2	100	100	91.7	3.4	4	13.6	14.2	13.8	0	0	8.3	3.4	41
2.3	100	100	100	100	89.9	98.0	2.0	2	0	0	0	0	10.1	2.0	2.0	100
2.4	100	79.3	77.9	77.5	79.1	82.8	4.3	5	0	20.7	22.1	22.5	20.9	17.2	4.3	25
2.5	100	88.6	100	88.0	88.3	93.0	2.9	3	0	11.4	0	12.0	11.7	7.0	2.8	40
3.1	89.7	100	89.7	90.1	100	94.0	2.5	3	10.3	0	10.3	9.9	0	6.1	2.5	41
3.2	100	100	100	90.3	100	98.1	1.9	2	0	0	0	9.7	0	1.9	1.9	100
3.3	100	100	81.3	81	100	92.5	4.6	5	0	0	18.7	19	0	7.5	4.6	61
3.4	100	88.6	100	100	89.2	95.6	2.7	3	0	11.4	0	0	10.8	4.4	2.7	61
3.5	100	100	100	0	100	80.0	20.0	25	0	0	0	0	0	0	0	0

Table S4. Variation of myrcene, limonene, camphene and bornyl acetate percentages in essential oil of *Picea abies* leaves during growing season, May–September. In Plant # column, the first numeral stands for habitat number (as described in Materials and Methods), the second – for individual plant number within that habitat (as in Figure 2); SD – standard deviation.

Plant	Myrcene				Limonene				Camphene				Bornyl Acetate			
#	Mean	SD	Min	Max	Mean	SD	Min	Max	Mean	SD	Min	Max	Mean	SD	Min	Max
1.1	1.81	0.14	1.62	2.00	14.26	4.76	11.31	22.72	12.12	2.77	7.74	15.18	20.82	1.92	18.79	23.74
1.2	3.34	0.58	2.54	3.92	19.85	1.76	16.85	21.37	11.64	2.56	8.91	15.26	17.17	7.80	10.25	28.27
1.3	2.32	0.42	1.96	2.94	15.39	6.69	11.62	27.28	13.55	4.14	10.28	19.44	26.39	5.53	20.12	31.57
1.4	3.91	2.05	2.12	7.41	15.32	9.78	8.51	32.58	10.90	4.11	4.17	14.21	11.39	1.89	9.79	14.50
1.5	2.48	0.23	2.26	2.82	19.58	7.33	15.63	32.57	11.44	3.44	7.69	15.67	14.25	4.94	7.68	21.19
2.1	1.56	0.55	0.74	2.24	15.56	4.78	7.83	19.52	4.80	1.36	3.16	6.83	39.42	13.41	21.40	50.88
2.2	5.20	2.28	3.87	9.27	16.70	9.07	11.98	32.90	11.66	1.90	9.06	14.24	23.90	5.10	15.05	27.75
2.3	5.90	1.72	5.02	8.96	29.18	3.41	27.18	35.26	9.08	1.89	5.72	10.32	20.96	2.24	17.30	22.90
2.4	3.89	0.42	3.32	4.40	15.80	4.36	12.61	23.31	10.43	2.44	7.67	14.27	20.97	4.32	16.33	27.66
2.5	2.70	0.82	1.32	3.49	15.52	6.80	6.51	25.42	8.10	2.81	3.53	10.44	23.59	7.17	16.27	33.48
3.1	2.62	1.52	1.08	5.10	14.01	8.86	6.11	29.20	8.37	4.55	2.71	13.20	22.93	6.26	15.73	29.99
3.2	3.74	0.54	3.03	4.29	18.96	2.13	16.06	21.51	10.21	3.95	3.66	13.39	21.93	3.13	18.06	25.63
3.3	3.26	1.62	1.49	4.66	9.11	1.76	7.79	11.08	7.42	3.72	3.35	11.05	16.30	1.88	14.59	18.40
3.4	2.66	0.44	2.23	3.34	17.19	6.33	12.37	26.67	10.33	2.73	5.80	13.23	25.46	6.88	13.99	30.55
3.5	1.49	1.02	0.00	2.54	11.16	7.86	1.34	21.98	3.12	2.25	0.00	5.84	22.70	5.41	14.79	27.77

Figure S1. Chiral-phase capillary GC analysis of (1S)-(–) and (1R)-(+) enantiomers of a-pinene in *Picea abies* (essential oil of individual plant No.1.1 in August).

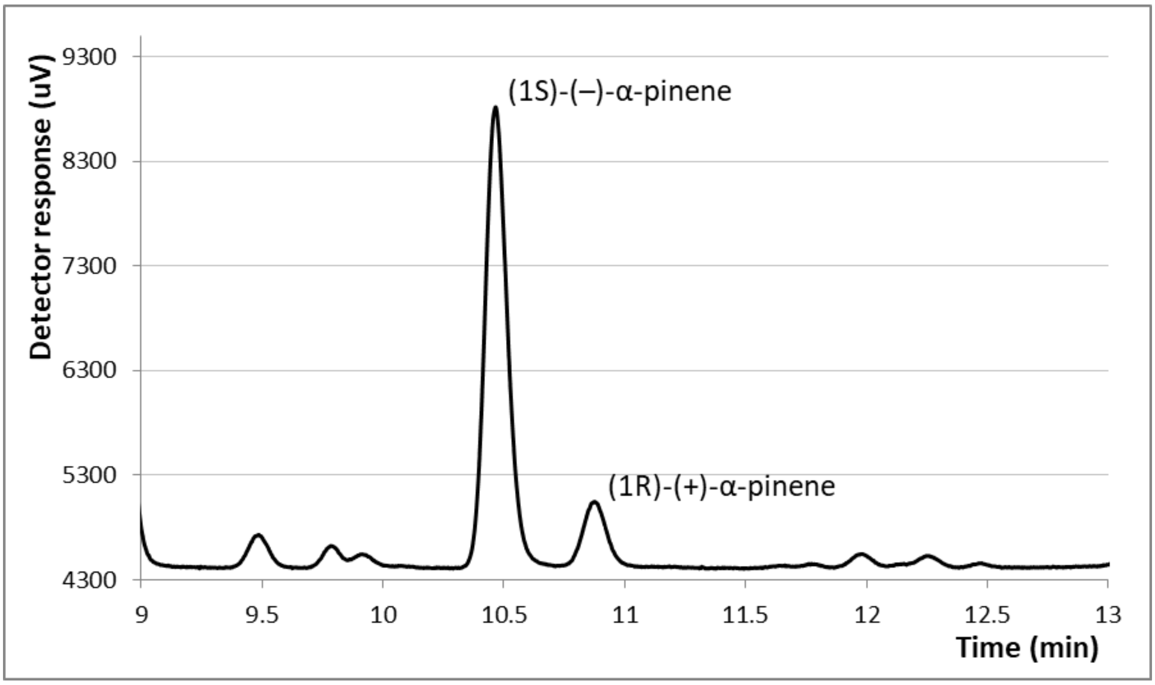

Supplement: Supplementary file 1 [file molecules-26-02138-s001.pdf]
